# Neuropilin-1 Knockout and Rescue Confirms Its Role to Promote Metastasis in MDA-MB-231 Breast Cancer Cells

**DOI:** 10.3390/ijms24097792

**Published:** 2023-04-25

**Authors:** Noura Al-Zeheimi, Yan Gao, Peter A. Greer, Sirin A. Adham

**Affiliations:** 1 Department of Biology, College of Science, Sultan Qaboos University, Muscat 123, Oman; n.alzeheimi@squ.edu.om; 2 Department of Pathology and Molecular Medicine, Queen’s University, Kingston, ON K7L 3N6, Canada; yg2@queensu.ca (Y.G.); greerp@queensu.ca (P.A.G.)

**Keywords:** neuropilin-1, knockout, breast cancer, metastasis, CRISPR, transcriptome

## Abstract

Breast cancer (BC) metastasis remains a leading cause of female mortality. Neuropilin-1 (NRP-1) is a glycoprotein receptor that plays ligand-dependent roles in BC. Clinical studies indicate its correlation with metastatic disease; however, its functional role in BC metastasis remains uncertain. CRISPR-Cas9 was used to knockout the *NRP-1* gene in MDA-MB-231 BC cells, and the effects on metastasis were determined using an orthotopic mouse engraftment model. *NRP-1* expression in knockout cells was rescued using a recombinant cDNA with a silent mutation in the sgRNA target-adjacent PAM sequence. Differentially expressed genes between *NRP-1* knockout and control cells were determined using whole-transcriptome sequencing and validated using real-time PCR. *NRP-1KO* cells showed a pronounced reduction in the metastasis to the lungs. KEGG pathway analysis of the transcriptome data revealed that PI3K and ECM receptor interactions were among the top altered pathways in the NRP-1KO cells. In addition, reduction in metastasis enhancers proteins, Integrin-β3 and Tenascin-C, and genes *CCL20* and *FN1* and upregulation of metastasis suppressor genes, *ACVRL* and *GPX3* in *NRP-1*KO were detected. These findings provide evidence for a functional role for NRP-1 in BC metastasis, supporting further exploration of NRP-1 and the identified genes as targets in treating metastatic BC.

## 1. Introduction

Locally advanced and metastatic disease are the leading causes of breast cancer patient mortality [1]. According to the global cancer statistics of 2020, breast cancer has surpassed lung cancer as the most diagnosed cancer in women [1]. Neuropilin-1 (NRP-1) is a widely expressed membrane cytoskeleton junction protein that has been implicated in promoting the growth and progression of different types of cancer through a variety of molecular mechanisms [2,3,4,5]; however, direct and functional roles in breast cancer metastasis have not been determined. Glycoprotein NMB (GPNMB)-driven increases in NRP-1 expression in BC cells were reported to potentiate vascular endothelial growth factor (VEGF) signaling and tumor growth but not metastasis [6]. Previously, we showed that elevation in circulating NRP-1 protein levels in the plasma of breast cancer patients is correlated with nodal and distant metastasis [7]. Similarly, another clinical study showed that NRP-1 protein concentrations in breast cancer tissue were significantly higher in patients with lymph node metastasis [8]. Furthermore, we showed that ectopic NRP-1 overexpression in BT-474 breast cancer cells promoted their in vitro pro-tumorigenic behavior [9]. Since breast cancer metastasis is the main cause of patient death [10], and the lungs are the primary site of metastases, particularly for patients with triple-negative breast cancer [11], we aimed to functionally inactivate NRP-1 in triple-negative breast cancer cells and study the impact on their metastatic potential.

Epithelial-to-mesenchymal transition (EMT) is a cellular dedifferentiation process by which cancer cells acquire invasive and metastatic behavior [12]. NRP-1/VEGFR signaling was shown to enhance breast cancer EMT through the activation of NF-κB and β-catenin in MDA-MB-231 cells [12,13]. A recent study showed that NRP-1 knockdown was associated with reduced proliferation in triple-negative claudin-low breast cancer cells and prolonged survival in a claudin-low orthotopic mouse xenograft model using MDA-MB-231 cells [14]. The lungs are one of the primary metastatic sites for this model [15], and several genes have been shown to either enhance or suppress BC metastasis to the lungs, but NRP-1 was not explored in these studies [16,17]. 

Evidence for the involvement of NRP-1 in tumorigenic behavior makes it a potential therapeutic target. Peptides targeting NRP-1 have been shown to induce apoptosis in human and murine breast cancer cells as well as endothelial cells, while a peptide targeting VEGFR2 only inhibited the growth of endothelial cells and did not affect the viability of breast cancer cells [18]. Tri-functionalized nanoparticles containing NRP-1 peptides, doxorubicin (DOX) and near-infrared cyanine dye (Cy7) have also been explored in in vitro and in vivo triple-negative breast cancer models [19]. These and other studies support the rationale of the present study, which uses genetic approaches to validate NRP-1 as a potential therapeutic target to suppress the metastatic behavior of triple-negative breast cancer.

## 2. Results

### 2.1. NRP-1 Knockout Dysregulates the TN-C/Integrinβ3 Axis and Downregulates HER-2

**Figure 1:**
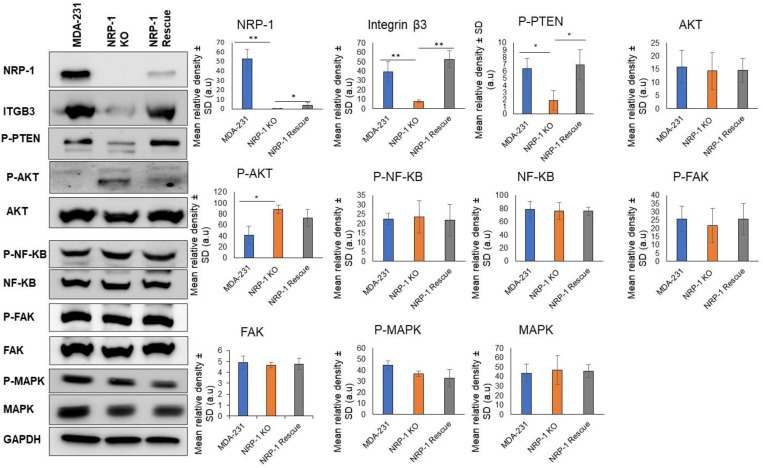
NRP-1 knockout in MDA-MB-231 cells was associated with a significant decrease in the levels of ITGB3 and P-PTEN. Western blot analysis of soluble cell lysates from parental MDA-MB-231 cells (MDA-231), an *NRP-1* knockout (NRP-1 KO) clonal cell line and a polyclonal rescue cell line (NRP-1 Rescue) of the NRP-1 KO cells. Blots were probed with the indicated antibodies, with GAPDH serving as a protein normalization control. The graphs represent the densitometry quantification of the protein bands from three independent experiments (*n* = 3), and the error bars represent mean ± SD. Values that differ significantly from the MDA-231 cells are indicated based on the *p*-value (* *p* < 0.05) (** *p* < 0.01).

We previously reported that the ectopic over-expression of NRP-1 in the HER-2+ breast cancer cell line BT-474 was correlated with an upregulation of the TN-C/Integrinβ3/ITGB3) axis [9]. Here, we further explored this relationship using the triple-negative breast cancer cell line MDA-MB-231. NRP-1 CRISPR-Cas 9 gene editing was used to knockout (KO) the *NRP-1* gene. *NRP-1* KO was validated in selected clones using Western blotting (Supplementary Figure S1a), and NRP-1 expression in selected *NRP-1* KO clones was rescued by subsequent lentiviral transduction with a CRISPR/Cas9-resistant *NRP-1* cDNA construct (Supplementary Figure S1a). Rescued KO clones displayed increasing levels of exogenous NRP-1 depending on the amount of rescue lentivirus used in transduction, and ~10% of parental cell endogenous NRP-1 protein levels were achieved at the highest transduction rate (Additional Figure S1a). Interestingly, *NRP-1* KO was associated with a decreased level of ITGB3 (*p* < 0.001) and P-PTEN (*p* < 0.001) but with an increase in P-AKT (P-S473). ITGB3 and P-PTEN levels were restored to parental levels in *NRP-1* rescue cells. There were no significant changes in total pan AKT levels, P-NF-kB/NF-kB, P-FAK/FAK, and P-MAPK/MAPK (Figure 1). 

**Figure 2:**
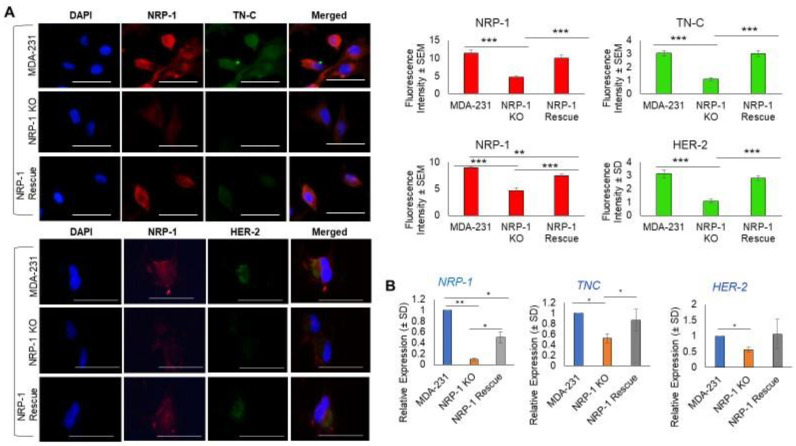
NRP-1 KO was associated with downregulation TNC and HER-2. (**A**) Dual immunofluorescence staining was performed to quantify the expression of the indicated proteins; NRP-1 in red, and TN-C or HER-2 in green in MDA-231, NRP-1 KO and NRP-1 rescued KO cells. DAPI staining localized the nuclei (left columns), and merged images are shown in the right columns. The graphs on the right represent the fluorescence intensities measured using Image J software, with data presented as mean values ± SD. ANOVA from summary data was performed and * *p* < 0.05 was the cut-off for significance. Scale bar = 100 µm. (**B**) Real-time PCR quantification results of NRP-1, TN-C, and HER-2 transcriptional levels were correlated with the protein expression results. (*) *p* < 0.05, (**) *p* < 0.01, (***) *p* < 0.001.

Since the ectopic overexpression of NRP-1 in BT-474 breast cancer cells was associated with the upregulation of tenascin C (TN-C) [9], we next explored this relationship in MDA -231, *NRP-1* KO, and NRP-1 Rescue cells using immunofluorescence staining (Figure 2, upper left). Both NRP-1 and TN-C measured fluorescence intensity was downregulated in *NRP-1* KO clones relative to parental control MDA-MB-231 cells, and both were restored in the NRP-1 rescued cells (*p* < 0.001). We previously observed that the ectopic overexpression of HER-2 in MDA-MB-231 cells was associated with an increase in NRP-1 levels [20]. Consistent with that previous report, we observed a reduction in HER-2 immunofluorescence staining in *NRP-1* KO cells, which was restored in the *NRP-1* rescued cells Figure 2 (lower left). (B) To confirm immunofluorescence (IF) data, Q-PCR was conducted, and the results were consistent with the IF data, the transcripts of *NRP-1*, *TNC*, and *HER-2* were all downregulated in NRP-1 KO cells. The mRNA levels of *NRP-1* in NRP-1 rescue cells were significantly different from NRP-1 KO and MDA-231 cells. *TNC* transcriptional levels were significantly lower in NRP-1 KO compared with MDA-231 and NRP-1 rescue. *HER-2* levels were significantly lower in NRP-1 KO when compared with control MDA-231 cells (Figure 2B).

### 2.2. NRP-1 Associations with ITGB3, and HER-2

**Figure 3:**
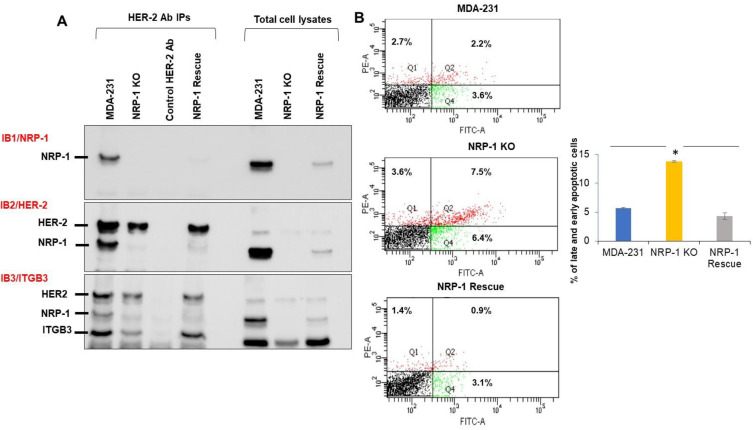
Immunoprecipitation and Western blotting confirm the protein–protein interaction between NRP1 and Integrinβ3 with HER2. (**A**) Western blot of HER-2 immunoprecipitation from cell lysates of MDA-231, NRP-1 KO, NRP-1 rescue, and control IP no lysate (control HER-2 Ab) was run beside the total cell lysates of the former cells. The blot was sequentially immunoblotted with the indicated antibodies (IB1 anti-NRP-1; IB2 anti-HER-2; IB3 ITGB3). (**B**) Flow cytometry analysis of apoptosis in serum-starved cells of the indicated genotypes was performed using Annexin V-FITC and PI staining of cells. Double Annexin V positive PI positive late apoptotic cells appear in quadrant 2 (Q2), while early apoptotic Annexin V positive PI negative cells appear in (Q4). The numbers indicated in the quadrants are the means of three independent experiments. The graph shows the average number of late and apoptotic cells; NRP-1 KO cells significantly increased in the sum of the late and early apoptotic cells when compared with parental and NRP-1 rescue cells, * *p* < 0.05.

We next explored potential direct or indirect interactions between NRP-1, HER-2, and ITGB3. HER-2 was immunoprecipitated from total cell lysates of the panel of MDA-MB-231 cells, and Western blotting was performed to first assess NRP-1 association (Figure 3A, IB1). NRP-1 was present in the anti-HER2 IPs from MDA-MB-231 parental and NRP-1 rescue cells in the same relative amounts as seen in the total cell lysates. No NRP-1 immunoreactive band was present in HER-2 IPs from the NRP-1 KO cells, nor could a band migrating in this position be seen in IPs using lysis buffer without any protein as a negative antibody control (control HER-2 Ab). Probing the same blot with anti-HER-2 antibody (IB2) confirmed the presence of HER-2 in the IPs. The same blot was then blotted a third time (IB3) with an antibody to ITGB3. We observed the same ratio of ITGB3 in the HER-2 IPs from all the cell lines as was apparent in the total cell lysates, suggesting the absence of NRP-1 did not affect the apparent association of ITGB3 with HER-2. This association of NRP-1 with HER-2 is consistent with our previously published observations [20]. 

### 2.3. NRP-1 KO Was More Sensitive to FBS Serum Starvation

Flow cytometry analysis was next used to assess the effect of NRP-1 on sensitivity to serum starvation (Figure 3B). The percentage of Annexin V-positive PI-negative early apoptotic cells and double-positive late apoptotic cells were significantly higher in *NRP-1* KO, indicating a greater sensitivity to serum starvation. This phenotype was reversed in the *NRP-1* KO rescued cells, supporting a role for NRP-1 in protection against serum-induced apoptosis (Figure 3B).

### 2.4. NRP-1 Knockout Did Not Impair Tumor Growth but Reduced Metastasis to the Lungs

**Figure 4:**
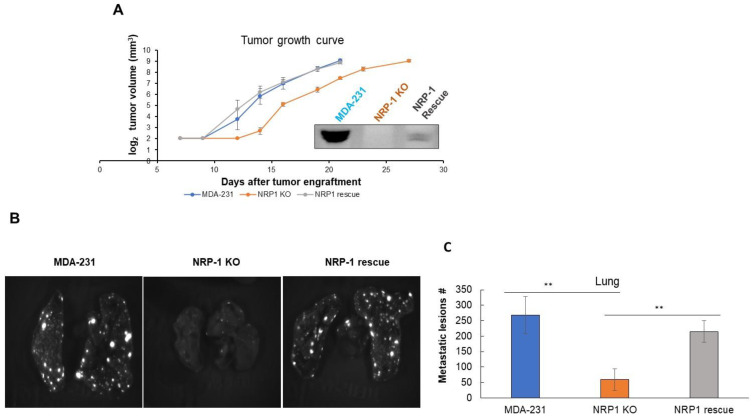
NRP-1 KO was associated with delayed tumor growth and reduced lung metastasis in a xenograft model of triple-negative breast cancer. MDA-MB-231 cells of the indicated genotypes (MDA-231, NRP-1 KO, and NRP-1 rescue) were engrafted into the mammary glands of *Rag2^−/−^; IL2R*γ*c^−/−^* female mice. (**A**) Tumor volumes were measured in mm^3^ and graphed on a log2 *y*-axis. The Western blot insert of the indicated cell lines illustrates the absence of NRP-1 in the NRP-1 KO and the restoration of reduced levels of NRP-1 in the NRP-1 Rescue cells relative to the parental MDA-231 cells. (**B**) Tumors were resected by recovery surgery at 600 mm^3^ volumes, and 10 days later, the lungs were resected, and metastatic lesions (white dots) were quantified by GFP detection from biophotonic images. (**C**) The graph illustrates the average number ± SD of lung lesions formed in the three cohorts (*n* = 6 mice per cohort; indicated *p*-values were calculated using the Wilcoxon rank sum test ** *p* < 0.01).

To explore the effect of NRP-1 knockout on the tumorigenic potential of these breast cancer cells, we next performed orthotopic xenograft analysis comparing tumor growth and the metastatic behavior of parental MDA-MB-231, *NRP-1* KO, and *NRP-1* KO rescued cells. The *NRP-1* KO cells gave rise to tumors that grew at the same rate as those from parental MDA-MB-231 and *NRP-1* KO rescued cells (Figure 4A). In some experiments, we observed a delay in the onset of tumor growth with the *NRP-1* KO cells, but this was not reproducible (Supplementary Figure S1b), so it may have been associated with different viability states of the cells at the time of engraftment.

To explore metastasis in these orthotopic engraftment experiments, we employed a resect-and-wait approach, where primary tumors were resected by recovery surgery when they reached endpoint volumes of 600 mm^3^, and the mice were maintained for ten days post-resection to allow distantly seeded metastatic lesions to grow to easily detected sizes. Resected lungs were then assessed by biophotonic imaging, and the total number of GFP-positive metastatic lesions were determined (white dots) (Figure 4B). This analysis showed that tumors from *NRP-1* KO cells gave rise to significantly fewer lung metastatic lesions than tumors from parental MDA-MB-231 cells, and this reduction was reversed by rescuing NRP-1 expression in the KO cells (Figure 4B). This rescued lung metastatic phenotype was reproduced in an independent experiment (Supplementary Figure S1b,c). 

Immunohistochemical staining of GFP in these lungs showed the expected invasive morphology of the metastatic MDA-MB-231 cells (Supplementary Figure S2).

### 2.5. Transcriptome Analysis Reveals Downregulation of ECM-Receptor Interactions in NRP-1 KO Cells

**Figure 5:**
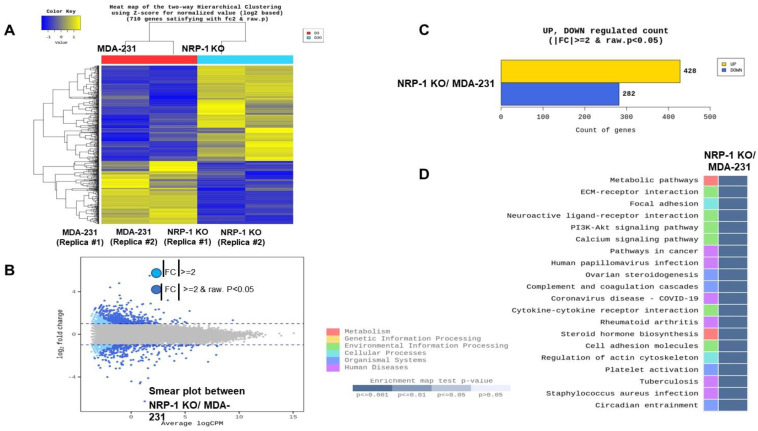
Total RNA sequencing was performed to decipher the transcriptional changes that might have reduced the ability of MDA NRP-1 KO cells to metastasize to the lungs. (**A**) The log10 (RPKM + 1) values of differentially expressed genes were used for cluster analysis. In the shown heat map, the upregulated genes are shown in yellow, and those with downregulated are shown in blue. (**B**) Differential expression of MA plot. Dark blue dots represent significantly upregulated genes, and light blue dots significantly downregulated genes. (**C**) Bar graph of genes significantly up- or downregulated between NRP-1 KO and parental cells. (**D**) Kyoto Encyclopedia of Genes and Genomes (KEGG) pathway enrichment of differentially expressed genes. Color coding indicates different value ranges of significance; the darker the blue squares indicate higher significance with the knockout of NRP-1.

**Figure 6:**
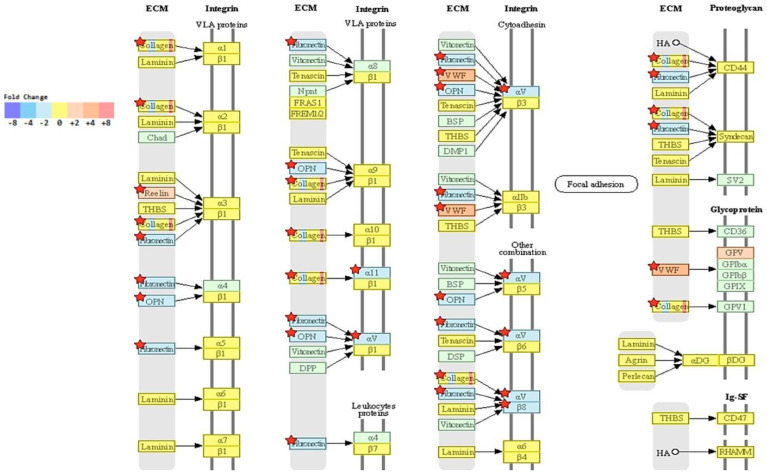
Significant suppression of the KEGG ECM pathway. Fibronectin and Osteopontin (OPN) were reduced, and the von Willebrand factor (VWF) increased in NRP-1 KO versus control cells. KEGG, Kyoto Encyclopedia of Genes and Genomes.

Since we focused on studying the proteins that were determined from the reciprocal of this study (i.e., recombinant expression of NRP-1 in BT-474 triple-positive breast cancer cells [9]. We explored the molecular effect of NRP-1 KO to gain insight into how NRP-1 KO affects global transcription behavior in the MDA-MB-231 cells. In addition, RNA and long non-coding total RNA sequencing and analysis were performed. Figure 5A shows that the normalized RNA-sequencing data from *NRP-1* KO and control cells (two different clones of each) were clustered separately using unsupervised principal component analysis; this resulted in a reproducible significantly different gene expression behavior. The result of hierarchical clustering (distance metric = Euclidean distance, linkage method = complete) analysis graphically represents the similarity of the gene expression patterns between samples. The highly expressed genes are labeled in yellow, while the low expressed ones are highlighted in blue. MA plot in Figure 5B shows the differential expression between the NRP-1KO and control cells. Light blue dots symbolize significantly downregulated genes, and dark blue dots represent significantly upregulated genes. The bar graph in Figure 5C shows the number of substantially up- or downregulated genes between the NRP-1 KO versus control cells. Four hundred twenty-eight genes were upregulated, and two hundred eighty-two were downregulated. The KEGG database was used further to determine the genes’ physiological activities and associated functions. KEGG pathway analysis and hypergeometric tests were performed to identify the pathways of the DEGs and related pathways that were significantly enriched compared to the transcriptome background. In Figure 5D, the heat map represents the KEGG pathway enrichment analysis, measured by the Rich factor, Q-value, and the number of genes enriched in these pathways. The top 20 KEGG pathways, most significantly enriched for the analysis, were selected and shown. One of the enriched KEGG pathways is shown in Figure 6, genes in red are upregulated, and those in blue are downregulated. Gene ontology (GO) enrichment analysis revealed that the NRP-1 KO was associated with a significant alteration in the extracellular matrix ECM–Receptor interaction and the PI3K signaling pathways. The analysis showed significant downregulation of tumor-promoting genes such as Fibronectin (*FN1*) and Osteopontin (*OPN*). The raw sequencing data are available upon the publication of this manuscript. 

### 2.6. Real-Time Polymerase Chain Reaction Validation of NGS Results 

**Figure 7:**
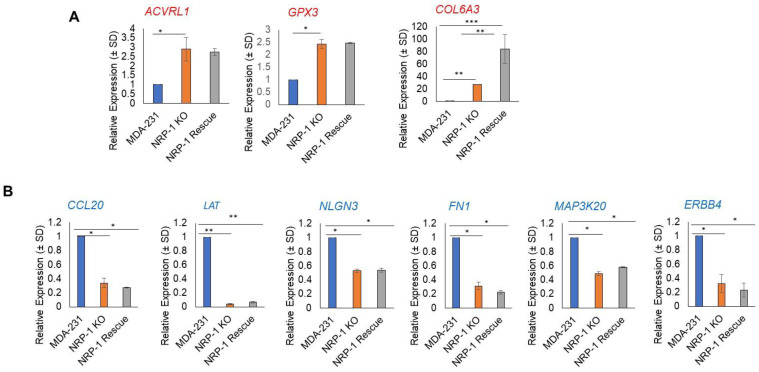
Quantitative real-time PCR validation of the up- and downregulated genes. From the selected 20 upregulated and 20 downregulated genes, three genes showed matching results with the RNA Seq data. The three upregulated genes are *ACVRL1*, *GPX3*, and *COL6A3*. The downregulated genes that matched with the RNA seq data are: *CCL20*, *LAT*, *NLGN3 FN1*, *MAP3K20*, and *ERBB.* The stars of the graphs represent the significant *p* values (* *p* < 0.05, ** *p* < 0.01, *** *p* < 0.001).

The significant DEGs between the control and NRP-1 KO from the total RNA sequencing analysis were selected on conditions of absolute fold change above or equal to 2 (|fc| ≥ 2) or fold change above 2 and less than 0.5, *p*-value < 0.05 per comparison and expression level above 2 for biological significance. Twenty upregulated and 20 downregulated DEGs were selected and quantified by qPCR, and the ones that showed matching results are shown in the graphs in Figure 7. Panel A shows the upregulated genes, and panel B shows the downregulated genes.

### 2.7. Lapatinib Treatment Caused a Decrease in P-AKT, P-FAK, and P-MAPK in NRP-1 KO Cells and Hindered the Cells’ Ability to Form Colonies

To further investigate the effect of NRP-1 knockout on downstream signaling, the cells were treated with Lapatinib inhibitors of EGFR and HER-2 [21]. The treatment with Lapatinib caused a decrease in the P-HER2, P- AKT, P-EGFR, P-FAK and P-MAPK. Notably, the latter two proteins’ phosphorylation was profoundly decreased in the NRP-1 KO cells. The ability of MDA- 231, *NRP-1* KO or NRP-1 rescue cells to form colonies was tested upon the treatment with Lapatinib. The cells were grown in monolayer cultures for 24 h with Lapatinib or untreated (vehicle control). The number of colonies formed in NRP-1KO and NRP-1 rescue cells was significantly less in Lapatinib-treated cells; however, control MDA-231 cells were not affected and produced a similar number of colonies as the vehicle control cells (Supplementary Figure S3a,b). 

## 3. Discussion

The lungs are a common metastasis site for breast cancer cells, and lung metastasis comprises one of the most common causes of breast cancer deaths [22]. Although several previous studies have reported anti-tumorigenic effects of targeting NRP-1 in different types of cancer [3,18,23,24,25], we are unaware of any reports on the involvement of NRP-1 in breast cancer metastasis to the lungs. In this report, we show that NRP-1 inactivation in MDA-MB-231 triple-negative breast cancer cells decreased lung metastasis. The observed reduction in the extracellular matrix (ECM) TNC protein (IF staining) and gene expression (qPCR) (Figure 2) in *NRP-1* KO cells may be related to this reduced metastatic behavior because it is a common and essential component of the lung metastatic niche [26]. Similarly, the NRP-1 knockout MDA-MB-231 cells displayed a significant decrease in the levels of integrin β3 (ITGB3), another extracellular adhesion protein well-known to enhance lung and bone metastasis of breast cancer cells once upregulated [27,28,29]. Indeed, ITGB1 and ITGB3 have been reported to play an important role in HER-2-induced mammary tumor growth and metastasis to the lungs, and this is associated with the promotion of signaling through the insulin receptor-AKT-mTORC1 pathway [29]. Crosstalk between NRP-1, ITGB3 and FAK was also found earlier in a mouse model, where the suppression of ITGB3 was associated with am NRP-1-dependent change in focal adhesion remodeling in endothelial cells and reduced tumor angiogenesis [30]. Although a previous study showed NRP-1 inhibition in glioma cells caused a decrease in the size of mice xenografts [31], our results showed no significant change in the rate of growth of the xenografts; although we did observe a delay in the onset of tumor growth in one of the replicas, this might be explained by the increase in phospho-AKT-S473 levels in the *NRP-1* KO cells (Figure 1), and the PI3K-AKT pathway was over presented in *NRP-1* KO based on RNA sequencing pathway analysis (Figure 5). While few functional studies have correlated NRP-1 expression directly with breast cancer metastasis to the lungs, we previously found that the plasma and tumor tissue expression of NRP-1 is significantly increased in advanced nodal and metastatic breast cancer compared with locally advanced disease [7]. Similarly, a clinical study on breast cancer tumor tissue indicated that NRP-1 concentration was significantly higher in patients with lymph node involvement compared with those without lymph node involvement [8]. A recent study found that miR-124-3p regulates NRP-1 expression in MDA-MB-231 cells, and reducing the expression of NRP-1 in mouse 4T1 cells reduced liver metastasis via the TGFβ pathway [32]. In prostate cancer PC3 cells, NRP-1 downregulation was correlated with the impaired ability of the cells to metastasize to the lungs upon nordihydroguaiaretic acid treatment [33]. 

In this study, NRP-1 expression in MDA-MB-231 cells correlated with their ability to produce tumors with metastatic potential in an orthotopic mouse xenograft model of metastatic triple-negative breast cancer. NRP-1 knockout was associated with an approximate 10x reduction in metastatic potential, and this was fully restored by rescuing NRP-1 expression in these KO cells. Transcriptome analysis of NRP-1 KO and control MDA-MB-231 cells revealed that the genes involved in PI3K and ECM receptor interactions were significantly altered. Gene Ontology terms related to biological processes showed that *NRP-1* KO was associated with a significant increase in genes involved in multicellular organismal processes, multicellular organism development, biological adhesion, and cell adhesion (Supplementary Figure S4a). In addition, gene ontology analysis indicated that genes regulating the cell periphery and plasma membrane are among the most altered genes in *NRP-1* KO over the control cells (Supplementary Figure S4b), which is consistent with our protein analysis results in which the plasma membrane proteins ITGB3, TN-C and, HER-2 were all downregulated in the *NRP-1* KO cells.

From the total RNA sequencing data analysis, the top upregulated and downregulated genes were selected and validated using quantitative real-time PCR. The qPCR results showed that *ACVRL1*, *GPX3*, and *COL6A3* genes were upregulated, consistent with the results obtained by RNAseq. *ACVRL1*, a member of the transforming growth factor β (TGFβ) receptor family, was overexpressed in NRP-1 KO cells. The overexpression of the *ACVRL1* gene in metastatic colorectal cancer patients treated with bevacizumab-based chemotherapy was shown to enhance progression-free survival and overall survival [34]. Glutathione peroxidase 3 (*GPX3*), the main antioxidant enzyme, was overexpressed in NRP-1 KO cells. Two reports on breast cancer showed that the overexpression of *GPX3* was negatively correlated with lymph node metastasis [35,36]. Additional studies in prostate cancer and thyroid cancer showed that the overexpression of *GPX3* reduces metastasis [37,38]. This might, in part, explain why NRP-1 KO cells showed fewer metastatic lesions in the lungs. Although Collagen Alpha-3 (VI) *COL6A3* was shown to enhance brain metastasis in breast cancer patients [39], *COL6A3* was upregulated in NRP-1 KO cells which showed less metastatic lesions to the lungs; future gain and loss of function studies will reveal more information about the actual role of this gene. 

On the other hand, *CCL20*, *LAT*, *NLGN3*, *FN1*, *MAP3K20*, and *ERBB4* genes were downregulated in NRP-1 KO cells, which might contribute to the decreased metastatic ability in the lungs. The downregulated C-C motif chemokine ligand 20 (*CCL20*) in NRP-1 KO cells was also studied in taxane-containing chemotherapy in triple-negative breast cancer (TNBC), and the elevated levels of *CCL20* were shown to stimulate breast cancer stem-like cells through protein kinase C (PKC) [40]. Another study conducted by Lee et al., 2017 confirmed the role of *CCL20* in breast cancer bone metastasis [41]. Therefore, it can be postulated that the reduction in CCL20 might play a role in decreasing the metastatic ability of the NRP-1/KO cells. The *LAT1* level in NRP-1 KO cells was also reduced and was shown to have higher levels in TNBC [42]. The overexpression of *LAT1* and CD98 in triple-negative breast cancer was a risk factor for relapse [42], and high levels of *LAT1* were correlated with poor prognosis in TNBC [43]. The neuronal-activity-regulated neuroligin-3 *NLGN3* was also downregulated in NRP-1 KO cells. Although no previous studies were found to correlate its expression to breast cancer metastasis, *NLGN3* was shown to advocate the growth of high-grade glioma [44,45]. Additionally, *FN1* which is an ECM remodeling-related molecule was downregulated in the NRP-1 KO cells and was shown to play a key role in breast cancer stroma invasion and metastasis [46]. *ERBB4* is a member of the HER-2 family and its knockdown activates apoptosis in lapatinib-resistant and trastuzumab-resistant breast cancer cells [47], indicating its role in tumor progression. Based on the KEGG analysis, ECM receptor interactions are on the top of the list for the most affected pathways significantly changed in the NRP-1 KO cells, supporting the need for further studies to explore the roles of each affected gene in lung metastasis.

The results we obtained in this and our previous studies support further exploring the strategy of targeting NRP-1 in breast cancer treatment, either with or without targeting the VEGF pathway since we showed that other genes can be targeted instead. Most of the previous studies depended on targeting NRP-1 synergistically with targeting VEGF for instance, in 2014, Patnaik et al. reported the results of a phase 1b clinical trial using a human monoclonal NRP-1 antibody MNRP-1685A in combination with bevacizumab in treating advanced solid tumors, the treatment caused higher than normal protein urea levels and, therefore, they decided to stop further testing of this antibody in combination with bevacizumab [48]. A very recent study reported the development of a new antibody, IDB0076, with a dual targeting ability for both NRP-1 and VEGFA, which showed promising preclinical efficacy [49]. In this study, we suggest looking at NRP-1 in a different context away from its correlation with VEGF but rather the other ECM receptors discovered from the big data obtained from this work.

## 4. Materials and Methods

### 4.1. Cell Culture

The human breast cancer cell line MDA-MB-231 was purchased from CLS Cell Lines Service, Germany, CLS, Cat# 300275/NA, RRID: CVCL0062). The cells were grown in monolayer cultures in a 5% CO_2_ incubator at 37 ^ο^C in DMEM (Sigma, Spruce Street, St. Louis, MO 63103, USA) supplemented with 5 mM of sodium pyruvate (Sigma, St. Louis, MO, USA), 10% FBS (Gibco, Grand Island, NY 1472, USA) and 2 mg/L of gentamicin (Gibco, Grand Island, NY, USA).

### 4.2. Knockout of the NRP-1 Gene in MDA-MB-231 Cells

A lentiviral-based CRISPR/Cas9 system was used to knockout *NRP-1* in MDA-MB-231 cells. Oligonucleotides were designed to encode a sgRNA that would direct Cas9 endonuclease activity to sequences within the 4th exon of the human *NRP-1* gene (sense: GGATGTTCTG TCGCTACGAC; anti-sense: GTCGTAGCGACAGAACATCC). These were cloned into the LentiCRISRPv2 vector (Add Gene plasmid #52961) and used to generate lentivirus, as described in [50]. Lentivirus-transduced MDA-MB-231 cells were selected for puromycin resistance, cloned, and subjected to Western blotting analysis to identify *NRP-1* null clones. The selected null knockout (KO) clones were transduced with pWPXLd (AddGene plasmid #12258) lentivirus-encoding GFP alone or GFP and a human rescue *NRP-1* cDNA with a silent mutation in the PAM element that follows immediately after the sgRNA target sequence. This mutation was introduced into the *NRP-1* cDNA using the QuickChange II XL Site-Directed Mutagenesis Kit (Agilent Technologies) according to the manufacturer’s instructions with the following mutagenic primer (GGATGTTCTGTCGCTACGACCGCCTAGAAATCTGGGATGG), where the bolded C residue corresponds to a mutation of the CG**G** PAM motif to a CG**C**. This silent mutation protected the rescue cDNA from CRISPR-Cas9-targeted disruption. Paired knockout WPXLd (KO) and rescue WPXLd/NRP-1 (R) cell lines were used for in vitro and in vivo experiments along with parental MDA-MB-231 cells.

### 4.3. Western Blotting

NRP-1 protein expression was assessed by using Western blotting. Briefly, the cells were washed with ice-cold PBS and incubated with lysis buffer for 1–2 min (Cat number 9803, Cell Signaling Technology, Trask Lane, Danvers, MA 01923, USA) in the presence of 0.1 mM phenylmethylsulfonyl fluoride (PMSF) protease inhibitor (Sigma-Aldrich Chemie GmbH, Eschenstrasse 5, D-82024 Taufkirchen, Germany). The protein cell lysate was vortexed and centrifuged for 20 min at 4 °C. The supernatants were collected, and protein quantification was carried out using DC Protein Assay (Biorad, 1000 Alfred Nobel Drive Hercules, CA 94547, USA). An equal quantity of protein lysates (100 μg of total protein per sample) were separated using 7.5% sodium dodecyl sulfate–polyacrylamide gel electrophoresis and then Trans-Blot Turbo Transfer System—Bio-Rad was used to transfer the protein onto polyvinylidene difluoride membranes (Biorad, 1000 Alfred Nobel Drive Hercules, CA 94547, USA). The immunoblots were then blocked with 5% non-fat milk protein in tris buffer saline-tween20 (TBST) buffer, shaken for 30 min at room temperature, and subsequently probed with rabbit primary monoclonal antibodies. All of the primary antibodies used and their sources are listed in Supplementary Table S1. GAPDH, used as a normalizing internal control, was detected with anti-GAPDH (Cell Signaling Technologies, Danvers, MA, USA, catalogue #2118) used at a 1:1000 dilution in blocking solution, and incubated at 4 °C overnight on a nutator platform. The blots were then washed three times for 5 min with TBST, followed by three washes for five minutes with TBS and incubated with goat anti-rabbit IgG horseradish peroxidase-conjugated secondary antibody (Abcam, Cambridge Biomedical Campus Cambridge CB2 0AX UK)at 1:5000 dilutions in blocking solution for 2 h at room temperature. The blots were then washed three times with TBST and three times for 5 min with TBS and developed using the clarity Western ECL substrate (BioRad, USA) according to the manufacturers’ instructions. The ChemiDoc touch imaging system (Biorad, USA) was used to visualize the protein bands. Densitometric analysis of ECL exposures was performed using the Image Lab software 4.4 (Biorad, 1000 Alfred Nobel Drive Hercules, CA 94547, USA).

### 4.4. Dual Immunofluorescence Staining

Cells were seeded on a sterile, positively charged slide in a tissue culture dish and allowed to attach overnight. The monolayer was then fixed with 4% paraformaldehyde, permeabilized using 0.05% Triton X-100, followed by blocking with 5% goat serum for 1 h at room temperature and overnight incubation at 4 °C degrees with a mixture of primary anti-rabbit NRP-1 antibody (1:100) (Abcam, UK) and anti-mouse TN-C antibody (1:200) (Santa Cruz Biotechnology, Inc. 10410 Finnell Street Dallas, Texas 75220 USA or a mixture of primary anti-rabbit NRP-1 antibody (1:100) (Abcam, Cambridge Biomedical Campus Cambridge CB2 0AX UK)) and anti-mouse HER-2 antibody (1:200) (Abcam, UK) diluted in PBS. After three washes with PBS at room temperature, the slides were incubated with Anti-rabbit IgG Fab2 Alexa Fluor 555 and anti-mouse IgG Fab2 Alexa Fluor 488 secondary antibody (Cell Signaling Technology, Trask Lane, Danvers, MA 01923 USA) at 1:400 dilutions in PBS for 1 h at room temperature degrees. After three washes with PBS at room temperature, the slides were counterstained with DAPI (1:250). Images were captured using a Nikon H600L fluorescent microscope (Japan) and NIS Elements software version 4.40. The fluorescence intensity of at least 40 cells per replica was measured using ImageJ software. 

### 4.5. In Vivo Tumor Growth and Metastatic Studies

GFP-expressing MDA-MB-231 cells of the indicated genotypes (1 × 10^6^ cells in 25 μL PBS/25 μL matrigel [Corning, VWR]) were injected into the inguinal mammary fat pads of 8–12-week-old *BalbC-Rag2^−/−^*; *IL2Rγc^−/−^* female mice. Seven days after engraftment, surgical staples were removed, and tumor volumes were assessed by caliper measurement every two or three days. When the tumors grew to 600 mm^3^, they were excised, and the mice were allowed to recover. Ten days later, the mice were euthanized for lung dissection and GFP-expressing metastatic nodules were imaged using a Hamamatsu B/W ORCA-ER digital camera with an excitation filter at 420 nm/20 nm and an emission filter at 520 nm/20 nm and metastatic lung lesions were manually counted. The mice were housed in the Animal Care Facility, and all procedures were carried out according to the guidelines specified by the Canadian Council on Animal Care with the approval of the institutional animal care committee.

### 4.6. Immunohistochemistry Staining of Lung Metastatic Lesions

Resected lungs were fixed in 10% formalin, processed and embedded in paraffin blocks, and 3 µm thick sections were processed on positively charged slides. After deparaffinization by serial incubations in xylene and ethanol (100%, 95%, and 70%), antigen retrieval was carried out by incubating the slides in 1 mM ethylenediaminetetraacetic acid (EDTA) pH 9.0 (Sigma-Aldrich) in a water bath at 95 °C for 30 min. After washing with PBS (Sigma-Aldrich, Germany), endogenous peroxidase activity was blocked using 2% hydrogen peroxide (H_2_O_2_) for 10 min. The sections were then washed with PBS, permeabilized in 0.05% Triton X-100 for 10 min, blocked in 5% goat serum (Dako, 5301 Stevens Creek Blvd, Santa Clara, CA 95051, USA) for 30 min, and then incubated with primary antibody (Anti-GFP antibody (#2956 cell signaling technology) at 4 °C overnight in a humidified chamber. The slides were then washed in PBS and incubated with the EnVision™ + Dual Link System-HRP (Dako, USA) labeled secondary antibody for one hour at room temperature, followed by incubation with substrate chromogen solution (DAB chromogen (Dako). The sections were counterstained using hematoxylin solution, dehydrated, mounted using DPX (Sigma, USA) and visualized using a (NikonH600L) light microscope. 

### 4.7. Co-Immunoprecipitation Analysis of NRP1 Binding to Integrin β3 to HER2

HER2 antibody was used to immunoprecipitate HER2 from soluble cell lysates prepared from parental MDA-MB-231, NRP-1 KO, or NRP-1 rescue cells using cell lysis buffer (Cell signaling technology Cat# 9803). Western blots were generated and immunoblotted sequentially with antibodies to NRP1(IB1 NRP-1), HER2 (IB2 HER-2) and Integrin β3 (IB3 ITGB3).

### 4.8. Colony Formation Analysis

The cells of the indicated genotypes (MDA-MB-231, NRP-1 KO, and NRP-1 rescue) were incubated for 24 h in 6-well plates with and without Lapatinib (abcam Cat number, 219408, Abcam, Cambridge Biomedical Campus Cambridge CB2 0AX UK) (14.9 µM in 0.087% DMSO). After the treatment period, the cells were trypsinized and counted using trypan blue seeded at a density of 1000 cells/well in 6-well plates (Corning, 5310 W. Camelback Rd., Glendale, AR, USA) and cultured for 14 days in the absence of drugs with two media changes during this period. The resulting colonies were washed with PBS, then fixed and stained with 5% crystal violet/methanol solution for 15 min at room temperature. This experiment was repeated independently three times.

### 4.9. Flow Cytometry Analysis of Apoptosis

The annexin V-FITC Apoptosis Detection Kit (Thermo Fisher, 5781 Van Allen Way Carlsbad, CA, USA 92008) was used to detect and quantify apoptosis by flow cytometry according to the manufacturer’s instructions. Briefly, 2 × 10^6^ cells were seeded in 100 mm dishes in a normal growth medium and cultured overnight. The next day, the cells were washed twice with PBS, and the media was changed to serum-free media and cultured for 24 h. The cells were then washed with PBS, detached using 1% trypsin, centrifuged, and resuspended in 500 μL of binding buffer and stained simultaneously with FITC-labeled annexin V (5 μL) and propidium iodide (PI) (5 μL). The cells were incubated at room temperature for 15 min in the dark before analysis by flow cytometry. A minimum of 10,000 events per sample were acquired on a FACS Aria III cytometer (BD Biosciences, GE Healthcare, 800 Centennial Avenue, Piscataway, NJ 08855-1327, USA) using the FITC detector for Annexin V-FITC and the PI detector. The data were analyzed using BD FACSDiva™ software.

### 4.10. Transcriptome Analysis

RNA sequencing was performed on MDA-MB-231 control and NRP-1 knockout cells in duplicate (a total of 4 paired-ends samples) to examine the different gene expression and ontology pathway analysis. Briefly, total RNA was extracted for library preparation using mRNA with poly-A tail and non-coding RNAs. The RNA was fragmented for short-read sequencing, which was then reverse transcribed to cDNA that was ligated with adaptors at both ends. The DNA fragments were amplified using PCR, and the fragments with insert sizes between 200 and 400 bp were selected for paired-end sequencing; both ends of the cDNA are sequenced by the full-read length method described in detail previously [51]. The quality of sequencing was verified using the phred score Q30 of the raw and trimmed data. The trimmed reads are mapped to the reference genome with HISAT2 version 2.1.0, Bowtie2 2.3.4.1. After the read mapping, StringTie was used for transcript assembly. The expression profile was calculated for each sample and transcript/gene as read count, FPKM (Fragment per Kilobase of transcript per Million mapped reads) and TPM (Transcripts Per Kilobase Million). DEG (Differentially Expressed Genes) analysis was performed on a comparison pair (NRP-1 Knockout_vs_Control) using DESeq2. The statistical method used was calculated based on fold change, nbinomWaldTest using DESeq2, and Hierarchical Clustering. Using each sample’s normalized value, the high expression similarities were grouped together. The significant results are selected on conditions of the absolute value of fold change |fc| >= 2 & nbinomWaldTest raw *p*-value < 0.05.

### 4.11. RNA Analysis by Quantitative RT-PCR (qPCR)

RNA was extracted from NRP-1 KO and control MDA-MB-231 cells using TRI reagent (Ambion, Austin, TX, USA) according to the TRIZOL protocol specifications. Prior to cDNA synthesis, RNA concentration and purity were measured by NanoDropTM 2000c spectrophotometer (Thermo Scientific, Waltham, MA, USA). Using a high-capacity reverse transcription kit (Thermo Fisher Scientific Baltics UAB | V.A. Graiciuno 8, LT-02241 | Vilnius, Lithuania), cDNA was synthesized from 1ug RNA and diluted at 5 ng/uL in DEPC-treated water and stored at −20 °C prior to gene quantification. The reaction mixtures for real-time PCR were prepared using Fast SYBR super mix (Applied Biosystem, Waltham, MA, USA); 15 ng cDNA and 100 uM of specific primers that were designed using Primer Express software (Primer Express, RRID:SCR_014326) (Applied Biosystems, Waltham, MA, USA). The primers and their specifications are listed in Supplementary Table S2. The 7500 Fast Real-time PCR system (Applied Biosystem, Waltham, MA, USA) was used to quantify the expression of selected differentially expressed genes (DEGs) under the following optimized conditions: enzyme activation at 95 °C for 20 s followed by 40 cycles of denaturing at 95 °C for 3 s and annealing/extension at 63.4 °C for 30 s (primer concentrations and annealing/extension times were optimized before targets quantification). Melting curves were plotted for each reaction. The Ct results generated from the system were analyzed using the 2^−ΔΔCt^ method to obtain the relative expression values.

## 5. Statistical Analysis 

All of the results are presented as mean ± standard deviation of the mean (SD). Statistical analysis was performed on the means of technical triplicates of at least three independently repeated experiments using a one-way ANOVA test for parametric data. Differences were considered significant when the *p*-values were less than 0.05. The stars of the graphs represent the significant *p* values (* *p* < 0.05, ** *p* < 0.01, *** *p* < 0.001).

## 6. Conclusions

Breast cancer cells that lacked NRP-1 failed to migrate to the lungs. The big data analysis revealed a set of pathways involved. However, the ECM receptors interactions came to the top of the list. Furthermore, protein analysis showed that part of the answer on how the cells lost their potential to metastasize to the lungs was due to the concomitant reduction in Integrin β3 and Tenascin C, well-known proteins involved in lung metastasis. In addition, the loss of NRP-1 from the cells made them sensitive to the HER-2 inhibitor and serum starvation. Finally, this report emphasized the importance of NRP-1 targeting with a new direction focusing on ECM proteins in treating metastatic breast cancer, which might aid not only in reducing the ability of the cells to metastasize but also in making them more susceptible to treatment. 

## Data Availability

The total RNA sequencing data discussed in this publication have been deposited in NCBI’s Gene Expression Omnibus and can be accessed at accession number GSE229031; https://www.ncbi.nlm.nih.gov/geo/query/acc.cgi?&acc=GSE229031.

## Supplementary Materials

The following supporting information can be downloaded at: https://www.mdpi.com/article/10.3390/ijms24097792/s1.
