# Supplementary material for: Neuropilin-1 Knockout and Rescue Confirms Its Role to Promote Metastasis in MDA-MB-231 Breast Cancer Cells
Source: Int J Mol Sci. 2023 Apr 25;24(9):7792. doi: 10.3390/ijms24097792 (PMC10178772; doi:10.3390/ijms24097792)
Supplement: Supplementary file 1 [file ijms-24-07792-s001.zip › ijms-2303941-supplementary.pdf]

NRP-1 Knockout and rescue in MDA-MB-231 cells

a

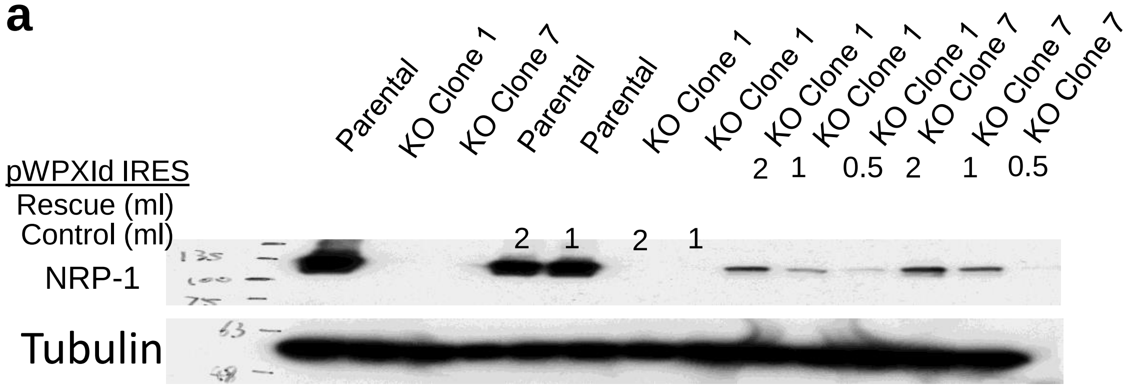

b

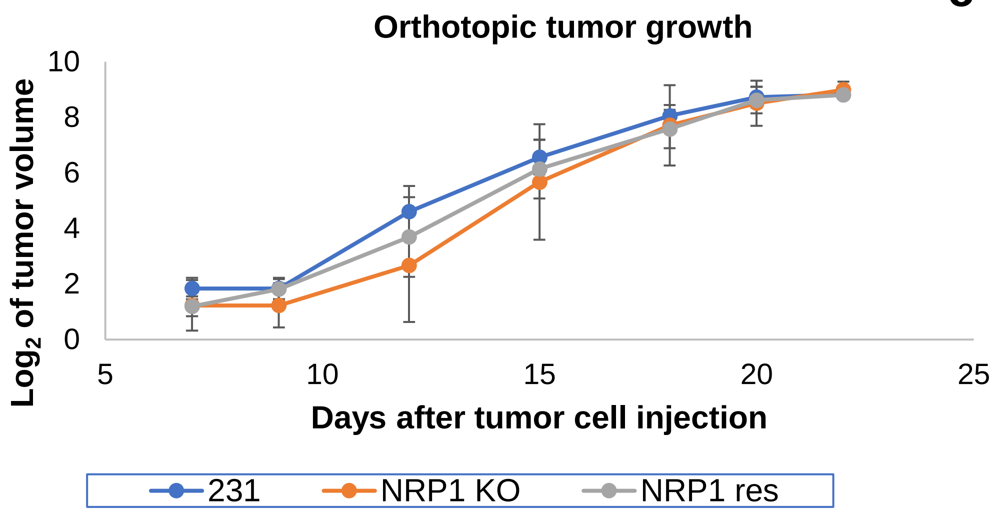

c

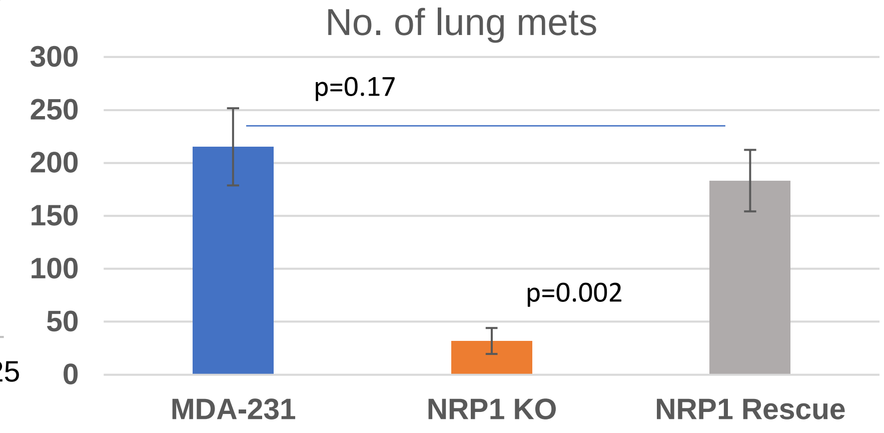

Figure S1. Additional Figure S1a. CRISPR-Cas 9 knockout and rescue of NRP-1 in MDA-MB-231 cells.
CRISPR-Cas 9 KO of the NRP-1 gene in MDA-MB-231 cells was carried out as described in the methods section. Clones with NRP-1 null phenotypes were identified by western blotting. Two of these were subjected to transduction with a lentivirus (pWPXLd IRES) encoding a CRISPR-resistant cDNA NRP-1 construct (Rescue), or the control empty vector (Control), in the indicated doses (0.5, 1 or 2 mL of virus-containing media). Immunoblotting for tubulin was used to assess protein loading.
Additional Figure S1b. Orthotopic xenograft tumor growth. One million MDA-MB-231 cells of the indicated genotypes (231, control parental), NRP-1 knockout (NRP-1 KO) or NRP-1 knockout with NRP-1 rescue (NRP-1 res) were engrafted into the mammary glands of Rag2-/-;IL2R c-/- mice, and tumor growth was monitored as described in the methods. Additional Figure S1c. Quantification of lung metastasis in an independent technical replica.

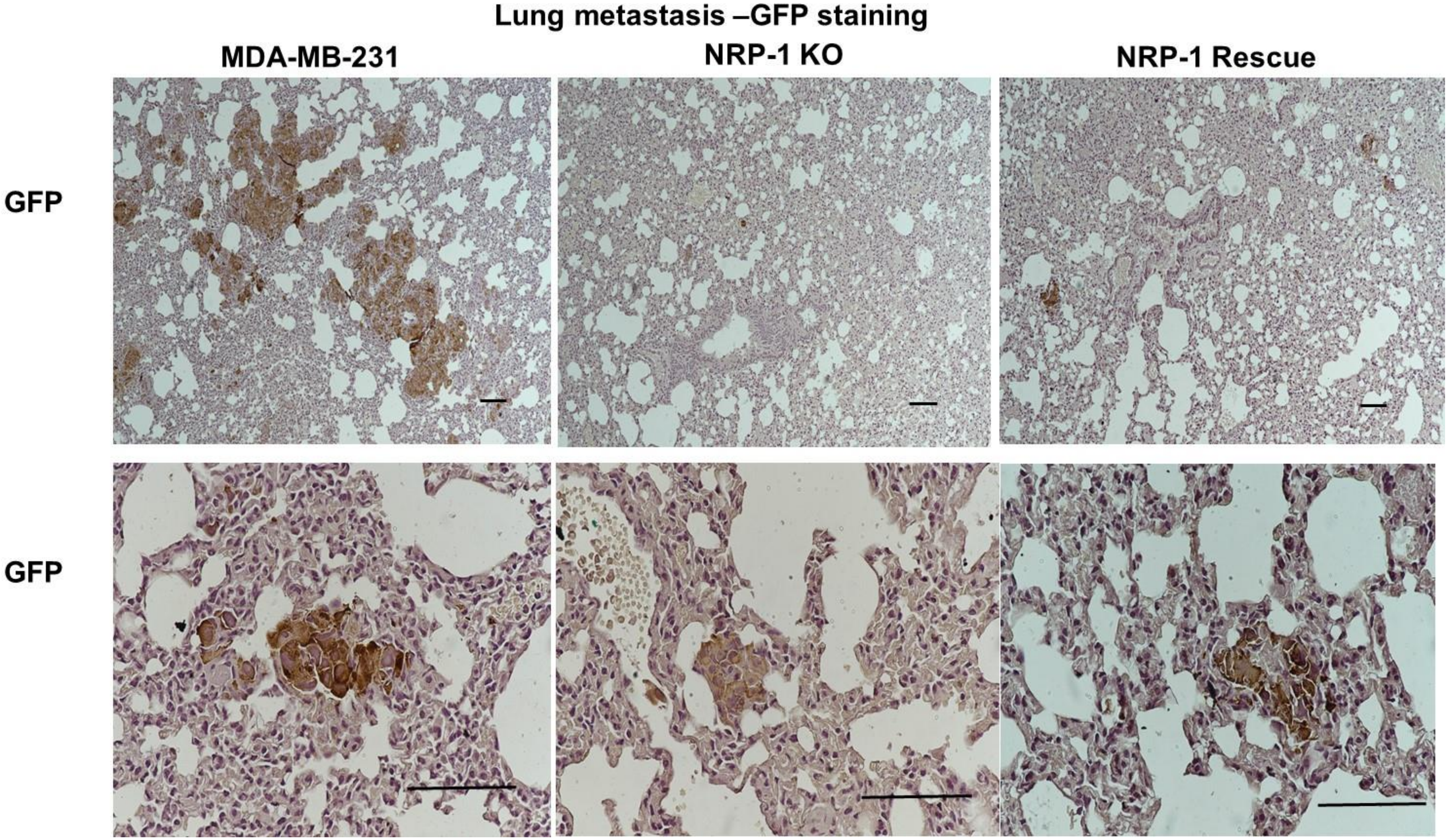

Figure S2. Immunohistochemistry staining of mice lungs stained with GFP. The MDA-231, MDA-KO, and MDA-rescue cells were localized in the lung tissues by GFP staining. Top panel, images taken using the 10 X objective and bottom panel the same images taken using the 20 X objective. The NRP-1 KO cells show the least number of metastatic lesions which was restored in the NRP-1rescue cells as indicated by the DAB brown color scale bar equals 100 µm.

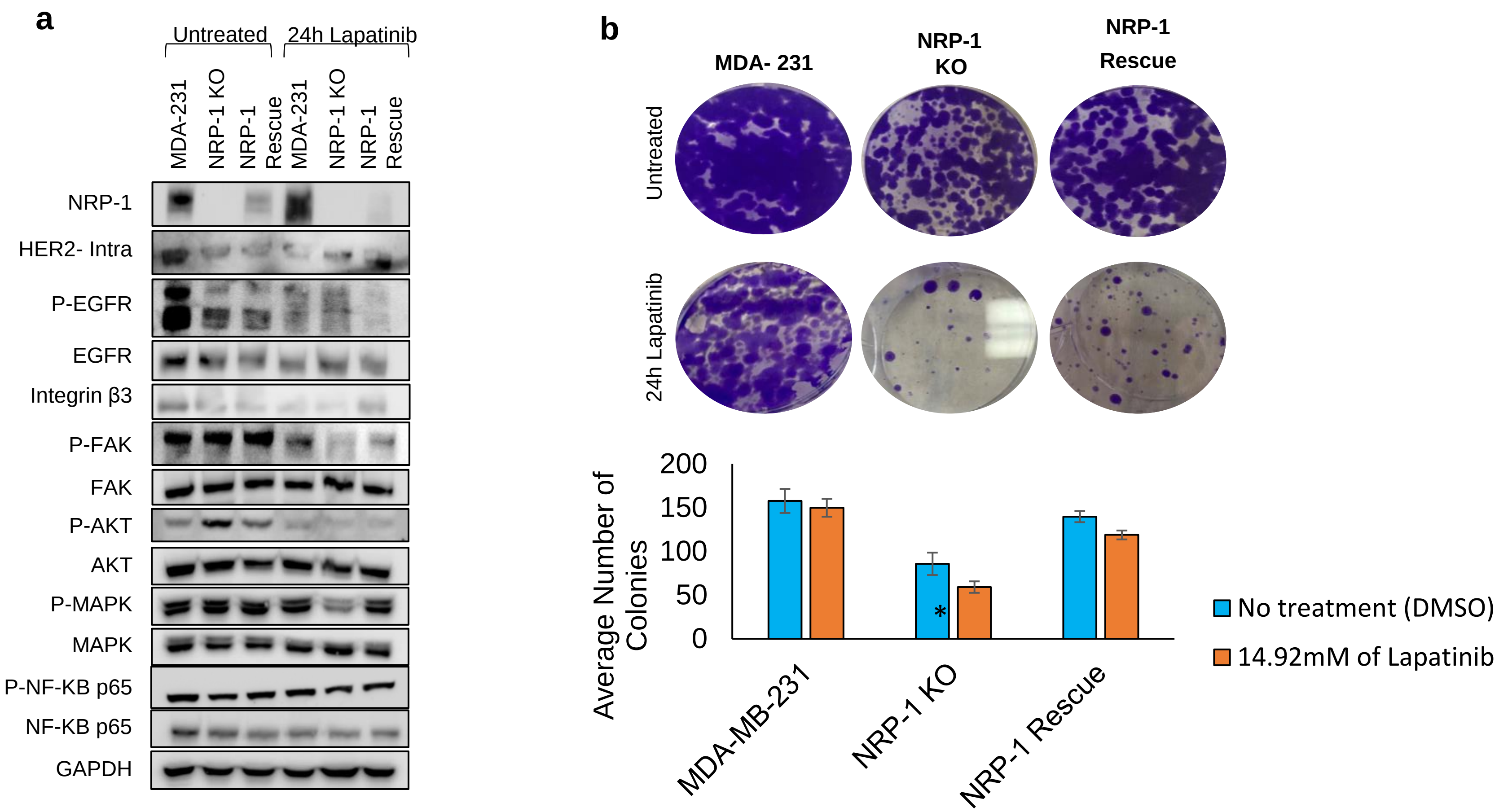

Figure S3. Lapatinib treatment in NRP-1 KO cells was associated with a decrease in the levels of P-AKT, P-FAK, P-MAPK and P-NF-KBp65 and reduced the ability of the cells to form colonies. (a) Western blot analysis of soluble cell lysates from parental MDA-MB-231 cells (MDA-231), an NRP-1 knockout (NRP-1 KO) clonal cell line and a polyclonal rescue cell line (NRP-1 Rescue) of the NRP-1 KO cells and soluble cell lysates from the three genotypes treated with lapatinib. Blots were probed with the indicated antibodies, with GAPDH as a protein normalization control. (b) Cells of the indicated genotypes were grown in monolayer cultures for 24 hours with lapatinib or untreated (vehicle control). The cells were then trypsinized, counted, and equal numbers of cells were replated at limiting dilutions in the absence of drugs. After colonies grew up, images of representative wells were taken after staining.

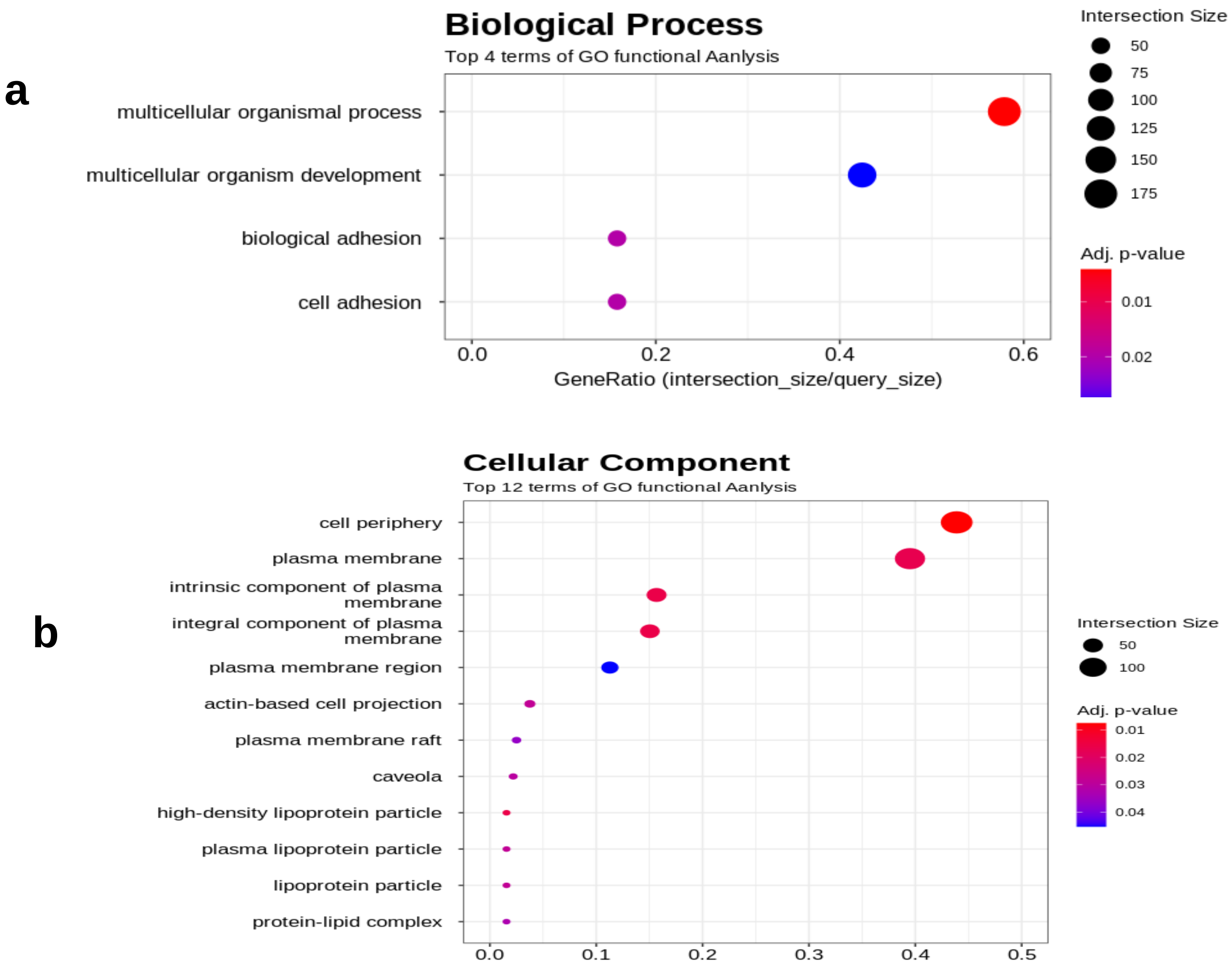

Figure S4. a & b. Gene ontology pathway analysis biological process and cellular component. The dot plot shows the results of the enrichment analysis based on Gene Ontology DB for significant genes.

Table S1: List of antibodies used for the western blots

Antibody	Cat. #	Manufacturer
GAPDH (glyceraldehyde-3-phosphate dehydrogenase)	5174	Cell signaling
NRP-1 (Neuropilin-1)	ab81321	abcam
p-NF-κB $_{P56}$ (Pospho-Nuclear factor kappa B)	3033	Cell signaling
NF-κB $_{P56}$ (Nuclear factor kappa B)	8242	Cell signaling
PI3K p110 α (Phosphatidylinositol-4,5-bisphosphate 3-kinase)	4249	Cell signaling
Phospho-PTEN (Ser380) Antibody (Phosphatase and tensin homolog)	9551	Cell signaling
p-Akt (S473)	4060	Cell signaling
Akt	4691	Cell signaling
HER-2/ErbB2 (29D8)	2165	Cell signaling
p-EGFR (Phospho- Epidermal growth factor receptor)	Her2/ErbB family Antibody Sampler kit (8339)	Cell signaling
EGFR (Epidermal growth factor receptor)	Her2/ErbB family Antibody Sampler kit (8339)	Cell signaling
FAK (Focal adhesion kinase)	ab40794	abcam
p-FAK (Pospho-Focal adhesion kinase)	ab81298	abcam
Integrin β3	13166	Cell signaling
Phospho-p44/42 MAPK MAPK p44/p42	9101	Cell signaling
p44/42 MAPK	4695	Cell signaling

Table S2: List of all primers used to amplify the transcripts in quantitative real time PCR.

Primers for quantitative real-time PCR
Col6A3 -F- 5’- CCCTCATCCAAAGCATCAAAG-3’
Col6A3 -R-5′-GTGTCTAAAGCAAAGGCTAGTTCTG-3′
ACVRL1-F-5″-TGGTGCTGTGGGAGATTGC-3′
ACVRL1-R-5′–CTATAGTCCTCCACGATGCCATT-3′
GPX3-F-5′ AACCCATGAAGGTTCACGACAT-3′
GPX3-R-5′ TGGCCCCACCAGGAACTT-3′
ETS1-F-5′GCCCTGGGTAAAGACTGCTTT-3′
ETS1-R-5′-CCCATAAGATGTCCCCAACAA-3′
CCL20-F-5′- ACCTCTGCGGCGAATCAG-3′
CCL20-R-5′- CTGTGTATCCAAGACAGCAGTCAA-3′
MAP3K20-F-5′-TCAAATGACACGAGCCTTCCT-3′
MAP3K20-R- 5′-TCCACTCCGCCTTGTTGTG-3′
LAT-F- 5′-GAGAGCGCAGAAGCGTCTCT-3′
LAT-R- 5′-TGCAGTTCCTGGGACACATTC-3′
NLGN3-F- 5’-GGCCTGGTCCAAATACAATCC-3′
NLGN3-R- 5′-GGTTTCAGCCCGATGTGAA-3′
FN1- F-5′- TCGCCATCAGTAGAAGGTAGCA-3′
FN1-R-5′- TGTTATACTGAACACCAGGTTGCAA-3′
ERBB4-F-5′- GCCTATTGTGGCAGAGAATCCT-3′
ERBB4-R-5′- GCACAGTGCCTGGCTTCAG-3′
NRP-1 F-5′-CCAGGTCGAATCCGATCCT-3′
NRP-1 R-5′- CGCTGTCGGTGTAAAAAACCA-3′
TNC F-5′-GGAACCAGGACAGGAGTACAATG-3′
TNC R-5′-CACGTGCGGGCTTGCT-3′
HER-2- F-5′-CCGTGCCACCCTGAGTGT-3′
HER-2-R-5′- AGCCTCCGGTCCAAAACAG-3′
IBTGB3-F-5′- GGACCCAGAGGACCAAGGAA -3′
IBTGB3-R-5′- GCAGGATGAAGCTGCCTACCT-3′
